# Pentoxifylline therapy among preterm neonates <1,500 g in reducing mortality from neonatal sepsis: a double-blind, randomized placebo-controlled trial

**DOI:** 10.1186/cc12914

**Published:** 2013-11-05

**Authors:** Jessamine C Sareno, Jacinto Blas V Mantaring

**Affiliations:** 1 Department of Pediatrics, Section of Newborn Medicine, Philippine General Hospital, Metro Manila, Philippines

## Background

Pentoxifylline, a xanthine derivative, has raised new interest in neonatal research due to its immunomodulatory functions and its potential role in reducing mortality from sepsis. Two small studies on a per-protocol analysis have shown promising results. This larger trial on an intention-to-treat basis will determine whether the use of pentoxifylline as an adjunctive therapy for sepsis in preterm neonates (≤36 weeks) weighing <1,500 g will truly result in a reduction in the all-cause mortality.

## Materials and methods

Preterm infants ≤1,500 g with suspected infection admitted to the NICU of a large tertiary, training, government hospital were eligible for inclusion in the study. After informed consent, they were randomized to receive either pentoxifylline at a dose of 6 mg/kg/hour or placebo. Patients with major congenital malformations, congenital infections and severe hemorrhage were excluded from the study. Pentoxifylline was administered as a 6 ml infusion for 6 hours for 6 days. The control group received normal saline in the same manner as the pentoxifylline infusion. Patients, parents and physicians (outcome assessors) were blinded to the treatment assignments. The primary outcome was analyzed on an intention to treat basis. The primary outcome measured in the study is the occurrence of all-cause mortality between the two groups. Secondary outcomes measured include mortality from sepsis, adverse drug reactions and length of hospital stay.

## Results

A total of 312 neonates are included in this interim analysis: 156 in the pentoxifylline group and 156 in the control group. Baseline characteristics were comparable between the two groups. In this analysis, there is no difference in the occurrence of death among patients in the pentoxifylline group versus the placebo group (RR: 1.08 (0.83, 1.41)). There is no statistical difference in the risk of death from septic shock (RR: 1.03 (0.67, 1.59), *P *= 1.0). There was also no significant difference in the length of hospital stay in the two groups (36 days in treatment group vs. 35 days in control group, *P *= 0.910). No significant adverse drug reactions were noted with pentoxifylline use.

## Conclusions

Pentoxifylline as an adjunct therapy for sepsis did not show a decrease in the all-cause mortality. There is also no difference in the occurrence of death from sepsis and length of hospital stay. No adverse drug reactions were noted with pentoxifylline.

